# Cancer-Associated Fibroblast Heterogeneity: A Factor That Cannot Be Ignored in Immune Microenvironment Remodeling

**DOI:** 10.3389/fimmu.2021.671595

**Published:** 2021-07-08

**Authors:** Pei-Yu Chen, Wen-Fei Wei, Hong-Zhen Wu, Liang-Sheng Fan, Wei Wang

**Affiliations:** Department of Obstetrics and Gynecology, The First Affiliated Hospital of Guangzhou Medical University, Guangzhou, China

**Keywords:** cancer-associated fibroblasts, heterogeneity, tumor immunology, immune evasion, tumor microenvironment

## Abstract

Cancer-associated fibroblasts (CAFs) are important, highly heterogeneous components of the tumor extracellular matrix that have different origins and express a diverse set of biomarkers. Different subtypes of CAFs participate in the immune regulation of the tumor microenvironment (TME). In addition to their role in supporting stromal cells, CAFs have multiple immunosuppressive functions, *via* membrane and secretory patterns, against anti-tumor immunity. The inhibition of CAFs function and anti-TME therapy targeting CAFs provides new adjuvant means for immunotherapy. In this review, we outline the emerging understanding of CAFs with a particular emphasis on their origin and heterogeneity, different mechanisms of their regulation, as well as their direct or indirect effect on immune cells that leads to immunosuppression.

## Introduction

The tumor microenvironment (TME) plays critical roles in tumor initiation, proliferation, and metastasis, and consists of cellular and extracellular matrix (ECM). Multiple cell types comprise the cellular compartment of the TME, including tumor cells, immune cells, endothelial cells, and cancer-associated fibroblasts (CAFs) (1). CAFs are activated in the tumor stroma, and formulate a highly heterogeneous group of cells with rich morphological characteristics and biological functions, which contributes to tumorigenesis by secreting growth factors, modifying the ECM, supporting angiogenesis, and suppressing anti-tumor immune responses (2–4). In this review, we summarize the various mechanisms concerning the immunosuppressive capabilities of activated fibroblasts in the TME, as well as their potential application for therapeutic intervention, especially in the field of tumor immunotherapy (5).

## Origin and Heterogeneity of CAFs

### Diversity of CAF Origin

CAFs constitute a unique cell population that significantly infiltrates the TME and contributes to the malignant phenotype and tumorigenesis. Emerging evidence suggests that CAFs have high heterogeneity. This could mainly be attributed to the numerous potential cellular sources of CAFs. It could be suggested that CAF heterogeneity may be partially explained by the fact that fibroblasts within one tumor can originate from different cellular precursors and from distinct cellular locations (6). CAFs can be derived from normal resident tissue fibroblasts that are activated by adjacent tumor cells. A majority of stromal activated fibroblasts initially originate from local resident fibroblasts, which respond to TGFβ and undergo myofibroblastic differentiation during tissue wound healing and cancer progression (7–9). Bone marrow-derived mesenchymal stem cells (MSCs) are a source of CAFs *via* recruitment by tumor cell secreted factors. BM-MSCs are upregulated Calponin 1, α-SMA and collagens by MRTF transcription factors to CAFs differentiation (10–13). Adipocyte conversion into CAFs has also been reported by several groups. Mature adipocytes activated Wnt/b-catenin pathway, leading to adipocyte “dedifferentiation” to acquire a fibroblast-like morphology (14–17). Endothelial cells are the potential source of CAFs which transfer by endothelial-to-mesenchymal transition (EndMT) in cancer. It has been shown that TGF-β2 induces the mesenchymal transdifferentiation of human microvascular endothelial cells to CAFs (18, 19). Evidence of pericytes converting into CAFs is relatively sparse, with tumorigenesis studies that specifically targeted pericytes not revealing large scale differences in the TME (20–22) (**Table 1**).

**Table 1:** The heterogeneity of CAF’s origins.

Origin	Markers	Functions		Refs
		Characteristic	Tumor-promoting	
Resident fibroblasts	α-SMA, type I collagen, CCL2、RAB3B, TNC	Form perivascular structures, Secrete TGF-β1	Angiogenesis, Migration, Metastasis	(7–9)
Bone marrow-derived MSCs	DDR2*, FSP-1, CXCL12, Vimentin, α-SMA, calponin-1, PDGFRα	Increases cancer cell growth and metastasis, Myofibroblastic differentiation	Proliferation, Migration, Invasion, Metastasis	(10–13)
Adipocyte	FSP-1, α-SMA, FAP, ASC-1	Contribute to the desmoplastic reaction, Increase tumor vascularization	Proliferation Angiogenesis	(14–17)
Endothelial cells	CD31, FSP-1, α-SMA, TGF-β2	Endothelial-to-mesenchymal transition	Angiogenesis, Proliferation, Invasion	(18, 19)
Pericytes	PDGF-BB, NG2^+^, FSP-1, α-SMA	Vascular remodeling toward a maturation phenotype	Invasion, Metastasis	(20–22)

*DDR2, Discoidin domain receptor 2.

### Phenotypic Heterogeneity of CAFs

The heterogeneity of CAFs is not only reflected in their origin, but can also be identified by diversity in biomarkers, subtypes, and functions. Several markers have been used to confirm the presence of CAFs. These include α-smooth muscle actin (α-SMA) (23), tenascin-C (24), neuron glial antigen 2, platelet-derived growth factor receptor-alpha/beta (PDGFR-α or β), fibroblast activation protein (FAP) (25), CD90, matrix metalloproteinase 2 (26), and podoplanin (27–29). In addition, CAFs are also positive for mesenchymal markers such as vimentin, type I collagen, fibronectin, FSP-1/S100A4 (30), and prolyl-4-hydroxylase. Nevertheless, none of these markers are specific to CAFs and can be expressed by other cell types; for instance, podoplanin is a well-known marker of lymphatic endothelial cells (31), α-SMA can be found in vascular muscular cells, and neuron glial antigen 2/PDGFRβ in normal pericytes. Furthermore, some types of normal stromal cells also express vimentin, fibronectin, type I collagen, prolyl-4-hydroxylase, and FSP-1/S100A4. The lack of specific markers is one of the most serious challenges in studying CAFs and manipulating them in their host tissues.

Although the extent of CAF heterogeneity remains undefined, CAFs are known to comprise a diverse cell population consisting of several subtypes. In other words, the lack of congruency in the marker expression raises the possibility that CAFs comprise a diverse group of cells made up of several subsets (32). Support for this notion comes from recent studies on a variety of cancers, such as pancreatic ductal adenocarcinoma (PDAC), breast cancer, colon carcinoma (33), oral carcinoma (34) and lung cancer (35), in which functionally distinct subclasses of CAFs were identified by various means based on the expression of a limited set of markers (20).

Research on the CAFs of PDAC has uncovered three CAF subsets. In the TME of PDAC, two distinct CAF subtypes characterized by either myofibroblastic or inflammatory phenotypes through transforming growth factor (TGF)-β and IL-1/JAK-STAT signaling as the major pathways have been identified (36). The myofibroblasts constitute a CAF subpopulation with an elevated expression of α-SMA located immediately adjacent to neoplastic cells in mouse and human PDAC tissues. They also described another subpopulation of CAFs as inflammatory fibroblasts, located more distantly from neoplastic cells, which lacked elevated α-SMA expression and instead secreted IL-6 and additional inflammatory mediators (37). Moreover, a new population of CAFs was termed as “antigen-presenting CAFs,” which express MHC-II and CD74, but do not express classic co-stimulatory molecules. They activate CD4^+^ T cells in an antigen-specific fashion in a model system, confirming their putative immunomodulatory capacity (38, 39).

According to different methods to mark CAFs in breast cancer research, various cell populations are birthed to diverse subtypes. A recent study demonstrated that a CD10^+^ G-protein-coupled receptor 77 positive subtype of CAFs promotes tumor formation and chemoresistance by providing a survival niche for cancer stem cells (35, 40, 41). Metastatic breast cancer axillary lymph nodes (LNs) exhibit four CAF subsets (CAF-S1 to -S4). In particular, CAF-S1 and CAF-S4 are the most abundant subsets in metastatic LNs, which correlate with cancer cell invasion. While CAF-S1 stimulates cancer cell migration and initiates an epithelial-to-mesenchymal transition through the CXCL12 and TGF-β pathways, the highly contractile CAF-S4 induces cancer cell invasion in three dimensions *via* Notch signaling (42–45). Another study showed that three distinct subpopulations of CAFs, defined as vCAFs, mCAFs, and dCAFs, apparently originate from a perivascular location, resident fibroblasts, and malignant cells, respectively (20) (**Table 2**). Furthermore, a fibroblast subset, expressed as a PDGFR, was identified as a marker for high-risk ductal breast carcinoma *in situ* (46).

**Table 2:** Heterogeneity of CAFs and therapeutic approaches.

Subsets	Markers	Signaling Pathways	Functions	Ref	Therapeutic Targets	
					Not Specific	Probably Specific
vCAFs	PDGFRα, Nidogen-2, Desmin, CD31	PDGF-CC signaling	Angiogenesis			novel targeted drugs
mCAFs	PDGFRα, Fibulin-1	PDGF-CC signaling	Immunosuppression	(20)		
dCAFs	PDGFRα, SCRG1	PDGF-CC signaling	Migration			
Myofibroblasts,	α-SMA^High^, FAP^+^, CTGF^+^, TNC^+^ TAGLN^+^	(TGF)-β and IL-1/JAK-STAT signaling	Migration, Invasion, Metastasis	(36–39)	CAR T-cell therapy, αFAP therapy, Oncolytic virus-based therapies,	Galunisertib, Losartan, Nab-paclitaxel
Inflammatory fibroblasts,	PDPN, IL-6, α-SMA^Low^, LIF	NF- kB signaling	Metastasis, Angiogenesis, Immunosuppression			–
Antigen-presenting CAFs	PDPN, Saa3, MHC-II gene, CD74	MTORC1 signaling	Immunosuppression			–
CD10^+^/GPR77^+^ CAFs	CD10, GPR77, IL-6	NF- kB signaling	Proliferation,	(35, 40, 41)	Vitamin D, Vitamin A, DNA vaccine	–
	α-SMA, PDGFRβ,		Migration,			
	FAP, FSP1, Collagen 1		Chemoresistance			
CAF-S1	FAP^High^, CD29^Med-high^, α-SMA^High^, PDPN^High^, PDGFRβ^High^	TGFβ-signaling, CXCL12 signaling	Proliferation, Migration, Invasion, Metastasis, Immunosuppression	(42–45)		αFAP therapy, Dasatinib, Galunisertib
CAF-S2	FAP^Neg^, CD29^Low^, α-SMA^Neg-Low^, PDPN^Low^, PDGFRβ^Low^	Not Describe	Not Describe			–
CAF-S3	FAP^Neg-Low^, CD29^Med^, α-SMA^Neg-Low^, PDPN^Low^, PDGFRβ^Low-Med^	Not Describe	Not Describe			–
CAF-S4	FAP^Low-Med^, CD29^High^, α-SMA^High^, PDPN^Low^, PDGFRβ^Med^	NOTCH signaling	Proliferation, Migration, Invasion, Metastasis			Dasatinib

### Diversity of CAF Functions

The activation state, stress response, and source of CAFs cause functional heterogeneity. CAFs promote tumor angiogenesis, ECM remodeling, and regulate the immune response, which is conducive to the exogenous process of tumor progression, and then indirectly play a pro-tumorigenic role. CAFs can effectively promote angiogenesis in tumor tissues by secreting a variety of pro-angiogenic factors such as vascular endothelial growth factor, fibroblast growth factor, and IL-6, and can also recruit endothelial progenitor cells into tumor tissues through stromal-derived factor-1 (SDF-1) to promote angiogenesis (47). CAFs degrade the extracellular stroma by releasing some matrix remodeling enzymes, such as matrix metalloproteinases, and promote the invasion and metastasis of tumor cells. By modifying the cytoskeleton, CAFs increase the hardness of the extracellular stroma and promote tumor progression to a certain extent (48).

In addition, CAFs may regulate drug resistance of tumor cells. For example, CAFs activate the JAK-STAT3 and PI3K-Akt pathways in breast cancer cells by secreting IL-6, inducing the upregulation of epithelial−mesenchymal transition and E3 ubiquitin ligase complex function, and targeting the degradation of estrogen receptor alpha through the ubiquitin proteasome pathway, thereby leading to tamoxifen resistance in breast cancer cells (49). Moreover, CAFs alter the structure and hardness of the ECM in a variety of ways to reshape the ECM, thereby preventing chemotherapies from reaching tumor cells.

Despite the powerful tumor-promoting effects of CAFs, some CAF subsets have been reported to have tumor suppressive functions, which further supports the concept of CAF heterogeneity in the TME. Rhim et al. reported that some components of the tumor stroma act to restrain tumor growth, and the reduction of stromal content by sonic hedgehog gene deletion led to the emergence of more aggressive tumors that exhibited undifferentiated histology, increased vascularity, and heightened proliferation (50). In addition, Liot et al. also demonstrated that the TME contains anti-tumor components (51). Although it is well known that the Wnt signaling pathway promotes tumor progression (52, 53), Green et al. proved that Wnt3a derived from CAFs can both promote and inhibit the growth of breast tumors (54). Another study reported that deletion of CAFs such as αSMA^+^ myofibroblasts in mice with PDAC increased the frequency of FOXP3^+^ regulatory T (Treg) cells, attenuated the extent of immune surveillance of the tumor, promoted the epithelial−to-mesenchymal transition and increased cancer cell invasion. In addition, CAF deletion failed to increase the efficacy of gemcitabine in PDAC and decreased mouse survival rate, indicating that α-SMA^+^ CAFs had an inhibitory effect on the tumor (55). The influence of CAF on tumorigenesis and development may be related to the tumor development stage and CAF phenotype induced in the TME. Brechbuhl et al. identified two subtypes of CAFs based on their CD146 expression. CD146^−^ CAFs conferred tamoxifen resistance to breast adenocarcinoma, whereas CD146^+^ CAFs reversed this phenotype and improve drug sensitivity (56).

In last few years, the diversity of CAFs origin, their phenotypic markers, and the function in the TME have gained a preliminary understanding. In conclusion, CAFs have diverse origins, including: tissue-resident fibroblasts, bone marrow-derived MSCs, adipocyte, endothelial cells, pericytes. Markers, like α-SMA, FAP, PDGFR-α or β, tenascin-C, FSP-1/S100A4, Podoplanin, vimentin, type I collagen, used to confirm the presence of CAFs. What is more, some CAF subsets were reported in succession: (a) vCAFs, mCAFs, dCAFs, (b) myofibroblasts, inflammatory fibroblasts, antigen-presenting CAFs, (c) CD10+/GPR77+ CAFs, (d) CAF-S1 to -S4. Concerning the functions of CAFs, on one hand, CAFs are capable of pro-tumor through ECM remodeling, proliferation, angiogenesis, immunosuppression, migration, invasion, metastasis, chemoresistance. On another hand, some specific subtypes may play an anti-tumor role. Therefore, clarifying the functions of different subpopulations may provide a basis for targeted therapies related to CAFs.

## Role of CAFs in Tumor Immunity

There are complex interactions between fibroblasts and immune cells in the tissue microenvironment. Since 1863, when Virchow first hypothesized that cancer develops as a product of unresolved inflammation (57), tumor-associated inflammation has been considered as a critical hallmark that shapes our modern understanding of cancer progression. It is now well established that inflammatory cells are indispensable participants in the neoplastic process, because they foster proliferation, survival, and metastasis (58). In the early stage of tumor development, both non-specific and specific immune mechanisms of the body can effectively prevent cells from transforming, proliferating, and eliminating abnormal cells. Despite the presence of a large number of immune cells in tumor tissues, with tumor progression, effective anti-tumor immune responses cannot be generated. In contrast, some immune cells are induced to play immunosuppressive roles (59). The tumor immune tolerance mechanism is involved in a variety of cells in the TME, among which CAFs can have direct and indirect effects on immune cells to participate in the TME immune response.

### CAFs Immunomodulate Antitumor Immunity

The immune system can recognize and destroy tumor cells based on tumor-specific antigens or stress-induced molecular expression. Immune cells play an important role in the immune system, which performs the immune response and function in the body; these include the T cells, which produce a specific immune response, and dendritic cells (DCs), which play a role in antigen presentation. However, CAFs affect the function of T cells and inhibit DCs through a variety of mechanisms, thereby directly suppressing the immune system and promoting tumor progression.

#### CAFs and Cytotoxic T Lymphocytes

A specific immune response is one of the most important means for the body to recognize and kill cancer cells, among which T cells, especially cytotoxic T lymphocytes (CTLs), have a direct killing effect on tumor cells as the main force of specific immunity (60). The human leukocyte antigen I and β2 microglobulin expressed on the surface of malignant tumor cells form a complex with specific antigen peptides. CD8^+^ T lymphocytes mainly bind to the complex through the surface T cell receptor to execute the killing function (61). The T cell receptors and associated signaling molecules aggregate at the center of the T cell/tumor cell contact region, forming so-called immune synapses and initiating a cascade that leads to the implementation of CTL effector function. CTLs destroy cancer cells directly through synaptic exocytosis of cytotoxic granules containing perforin and granzymes, or indirectly, through the secretion of cytokines, including interferon γ and tumor necrosis factor (60, 62).

It has been shown that CAFs participate in tumor immunosuppression by inhibiting T cell migration and infiltration. Chen et al. found that blocking the CXCL12/CXCR4 signaling pathway in CAFs reduced the fibrosis level in a mouse model of metastatic breast cancer, improved the infiltration of CTLs, and reduced the immunosuppressive effect, thus improving the effect of immunotherapy (63). TGF-β signal transduction in CAFs weakens the effect of anti-PD-L1 drugs by controlling T cell migration. TGF-β blockers combined with anti-PD-L1 antibodies inhibit TGF-β signal transduction in CAFs, which facilitates the infiltration of T cells into cancer nests and stimulates effective anti-tumor immunity (64–66).

**Figure 1:**
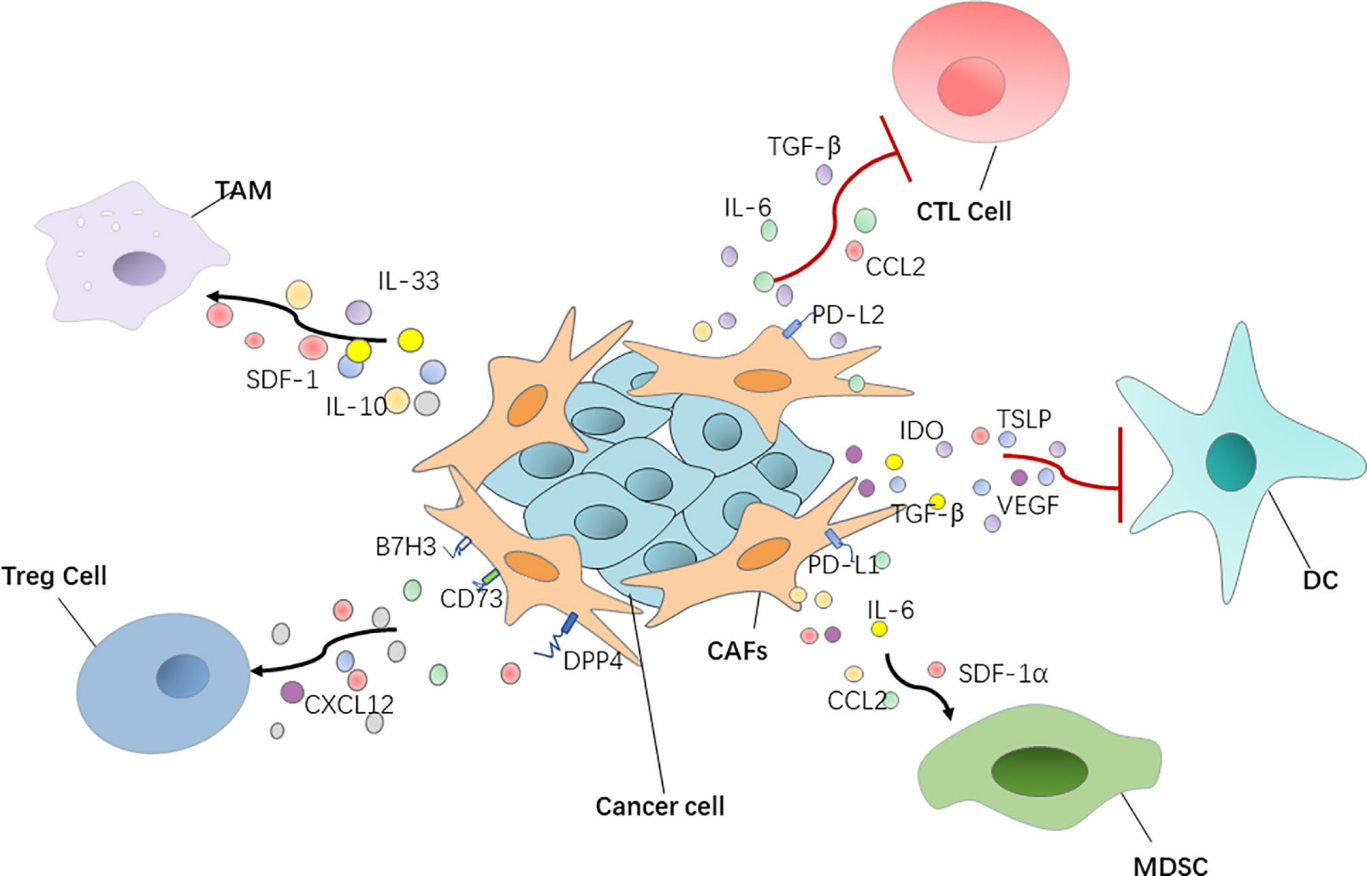
CAFs have a positive or negative interaction with immune cells *via* regulating various cytokines and chemokines. DC, dendritic cell; Treg cell, regulatory T cell; MDSC, myeloid‐derived suppressor cell; TGF‐β, transforming growth factor‐β; SDF-1, stromal-derived growth factor-1.

Furthermore, CAFs secrete a variety of cytokines to directly affect T cell function. Chen et al. demonstrated that CAFs with high FAP expression promote immunosuppression in the colorectal cancer TME by upregulating CCL2 secretion, recruiting myeloid cells, and decreasing T cell activity (67) (**Figure 1**). Takahashi et al. showed that stromal βig-h3 protein, which is a 68 kDa ECM protein, also known as TGF-βi, is highly expressed by CAFs in pancreatic cancer and acts directly on tumor-specific CD8^+^ T cells by reducing their proliferation and activation (68). Cheng et al. reported that CAF-derived IL-6 induces PD-L1^+^ neutrophils *via* the JAK-STAT3 pathway, which impairs T cell function through PD-1/PD-L1 signaling, and then creates optimal conditions for human hepatocellular carcinoma progression (69) (**Figure 1**). As was mentioned above, MHC-II expressed by antigen-presenting CAFs acts as a decoy receptor to deactivate CD4^+^ T cells by inducing either anergy or differentiation into Treg cells (38).

Furthermore, CAFs induce T cell death through the modulation of antigen presentation and expression of co-inhibitory receptors. Gorchs et al. found that human pancreatic CAFs promote the expression of co-inhibitory markers in CD4^+^ and CD8^+^ T cells. Functional assays showed that proliferating T cells expressing co-inhibitory receptors produced less interferon-gamma, tumor necrosis factor-alpha, and CD107a after re-stimulation when CAFs were present. This indicates that CAFs induce the expression of co-inhibitory receptors on CD4^+^ and CD8^+^ T cells, which contributes to the diminished immune function (70). CAFs sample, process, and cross-present antigens, killing CD8^+^ T cells in an antigen-specific manner *via* PD-L1 and FASL. The coincident antigen-specific upregulation of FAS/FASL and PD-1/PD-L2 on T cells and CAFs, respectively, drives the death and dysfunction of tumor-specific T cells, resulting in enhanced tumor viability (71). Additionally, Takahashi et al. showed that CAFs expressed the co-regulatory molecules B7H1 and B7DC whereas normal fibroblasts did not. B7 protein family members B7H1 and B7DC, expressed on the CAF surface, bind to PD-1 to activate T cells; these proteins are putative negative regulators of immune function and preferentially induce T cell apoptosis (72).

#### CAFs and Dendritic Cells

DCs are important antigen-presenting cells in the body and are responsible for the activation of T cells against foreign antigens, as well as regulating the immune response against self-molecules. Conditioning DCs with fibroblasts triggers them to express higher levels of co-inhibitory molecules and anti-inflammatory cytokines and lower levels of pro-inflammatory cytokines. Fibroblast conditioning arrests the ability of DCs to induce T cell proliferation *via* both direct and indirect pathways (73).

Cheng et al. showed that CAF-derived indoleamine 2, 3-dioxygenase and vascular endothelial growth factor inhibited the maturation and antigen uptake activity of DCs, thus inducing a tolerogenic phenotype in T cells (74). Weber et al. reported that TGF-β secreted by CAFs can influence biological properties of DCs and inhibit their ability to migrate, thereby hindering the process of antigen transport and presentation (75). In addition, thymic stromal lymphopoietin, which favors Th2 cell polarization through myeloid DC conditioning, is released by activated CAFs, inducing the activation and maturation of tumor antigen-loaded resident DCs. Activated DCs migrate to the draining LNs where they activate tumor antigen-specific CD4^+^ Th2 cells to exert tumor-promoting effector functions (76) (**Figure 1**).

### CAFs Facilitate Immunosuppressive Cells

In the TME, there is a large number of immunosuppressive cells, which have negative immunomodulatory functions, and play important roles in the invasion and metastasis of tumors, including Treg cells, myeloid-derived suppressor cells (MDSCs), and tumor-associated macrophages (TAMs). CAFs not only promote the differentiation and formation of these inhibitory cells, but also secrete a variety of chemokines to immunosuppressive cells to aggregate in tumor tissues and indirectly exert an inhibitory effect on the immune system.

#### CAFs and Regulatory T Cells

Treg cells, which suppress aberrant immune responses against self-antigens, also suppress the anti-tumor immune response. The infiltration of a large number of Treg cells into tumor tissues is often associated with poor prognosis (77). CAFs can play an immunosuppressive role by affecting Treg cell function. Human ovarian fibroblasts treated with boromycin, hydrogen peroxide, or radiation have been reported by Shen et al. to secrete WNT16B, which promotes the secretion of IL-10 and TGF-β by DCs, thus leading to the proliferation of Treg cells (78). In high-grade serous ovarian cancers, researchers uncovered four different CAF subpopulations, including CAF-S1 and CAF-S4, which express high levels of polymerized SMA. In particular, the CAF-S1 subset expressing high levels of CXCL12β, through a miR-141/200a dependent mechanism, increases the attraction, survival, and differentiation of CD25^+^ FOXP3^+^ T cells to exert immunosuppressive functions (79). In breast cancer, Costa et al. reported that CAF-S1 cells enhance regulatory T cell capacity through high expression levels of B7H3, DPP4, and CD73 (45) (**Figure 1**).

#### CAFs and Myeloid-Derived Suppressor Cells

CAFs have an ability to recruit MDSCs, of which the main feature is their potent immune suppressive activity. Yang et al. showed that CAF STAT3-CCL2 signaling in this setting promoted human intrahepatic cholangiocarcinoma growth by enhancing recruitment of MDSCs (80). CAF-derived cytokines, such as IL-6 and SDF-1α, can induce MDSC generation and activation and impair human anti-tumor immune responses to promote hepatocellular carcinoma progression (81). In liver cancer in particular, CAF-derived IL-6 can recruit myelinated inhibitory cells and upregulate the expression of PD-L1, thus reducing the immunotherapeutic effect of anti-PD-L1. When anti-PD-L1 is combined with anti-IL-6, the response of tumor cells to the PD-L1 treatment can be improved (82). CAFs secreting SDF-1α, which can recruit MDSCs to the TME to exert tumor-promoting effects in estrogen receptor-positive breast cancer, has also been reported (83). CAFs with high FAP expression recruit myeloid cells by upregulating CCL2 secretion to promote immunosuppression in the colorectal cancer tumor immune microenvironment (67) (**Figure 1**).

#### CAFs and Macrophages

Macrophages are widespread immune cells in the body, and they represent an important part of innate immunity. They play an important role in maintaining tissue stability, promoting tissue repair and regeneration, and participate in various pathophysiological processes, such as wound healing, inflammation, and tumor emergence and development (84). Takahashi et al. found that CAFs promote the induction and accumulation of pro-tumoral macrophages through the upregulation of CD80 and CD86 (85). In addition, CAFs not only recruit monocytes, but can also induce these recruited monocytes to differentiate into PD-1 expressing anti-inflammatory macrophages, rather than into pro-inflammatory macrophages. In other words, the PD-1 axis might be crucial in CAF-induced immune suppression *in vivo* in several types of cancers, including breast and colorectal tumors (86). Additionally, CAFs are also able to induce the macrophages to play the function of immunosuppression by producing anti-inflammatory cytokines IL-10 and IL-33 and decreasing the production of the pro-inflammatory cytokine IL-12 (87, 88). Recent data showed that CAFs, through SDF-1 secretion, promote anti-inflammatory macrophage expression and prostate cancer progression (89) (**Figure 1**).

## Therapeutic Targeting of CAFs

Traditional cancer treatments, including chemotherapy and radiation, target cancer cells, especially those that are growing actively. However, efficacy of these therapies varies greatly, so drug resistance, recurrence and metastasis of tumors remain a great challenge in clinical practice, suggesting the complexity of the mechanisms behind tumor evolution and heterogeneity. TME is a key factor affecting the evolution and heterogeneity of tumors. Targeting TME may provide a new strategy for the treatment of tumors. CAFs exert tumor immunosuppression in the TME during cancer progression, which makes them promising therapeutic targets for cancer intervention. Over the last several years, as understanding of CAF biology in cancer progression has steadily increased, there has been considerable interest in CAF-targeted therapies, and a number of preclinical studies have been carried out.

FAP, the main marker of CAFs, also plays an important role in tumor immunosuppression. Recently, adjuvant therapies targeting CAFs that express FAP molecules on their surface have been implemented in some animal models. Fang et al. significantly inhibited the growth of breast cancer tumors in mice by a combination of FAP^+^ CAF abrogation and treatment with paclitaxel. In the future, anti-CAF strategy may be combined with chemotherapy or other treatments to weaken the therapeutic resistance caused by the TME remodeling to achieve better efficacy (90). Zhen et al. reported that a nanoparticle-based photoimmunotherapy approach can be used to selectively kill CAFs without causing systemic toxicity. Specifically, they exploited ferritin, a compact nanoparticle protein cage, as a photosensitizer carrier and conjugated the FAP-specific single chain variable fragment to the ferritin surface. The administration of the nanoparticles suppressed CXCL12 secretion and ECM deposition upon photoirradiation, leading to significantly reduced the immunosuppression, enhanced T cell infiltration, followed by efficient tumor suppression (91). The modified synthetic consensus FAP DNA vaccine synergized with other tumor antigen-specific vaccine therapies in tumor-bearing mice (92). Moreover, adoptive transfer of FAP-specific chimeric antigen receptor to T cells proved to be effective in restraining tumor growth in preclinical models (93). In addition, FAP-targeting oncolytic adenovirus in tumor-bearing mice enhanced anti-tumor immunity by activating endogenous T cells to attack FAP^+^ stromal cells. Sostoa et al. used FAP-targeting bispecific T-cell engager (FBiTE) inserted in the ICOVIR15K oncolytic adenovirus under the control of a major late promoter to direct tumor infiltrated lymphocytes against CAFs. FBiTE-mediated binding of CD3^+^ effector T cells and FAP^+^ target cells led to the activation and proliferation of T-cells as well as their cytotoxicity toward FAP-positive cells *in vitro*. *In vivo*, FBiTE expression increased intratumoral accumulation of T cells and decreased the level of FAP in tumors. Combination of viral oncolysis of cancer cells and FBiTE-mediated cytotoxicity toward FAP-expressing CAFs might be an effective strategy to overcome the barrier of the tumor-associated stroma, a key obstacle to oncolytic virotherapy (94).

**Figure 2:**
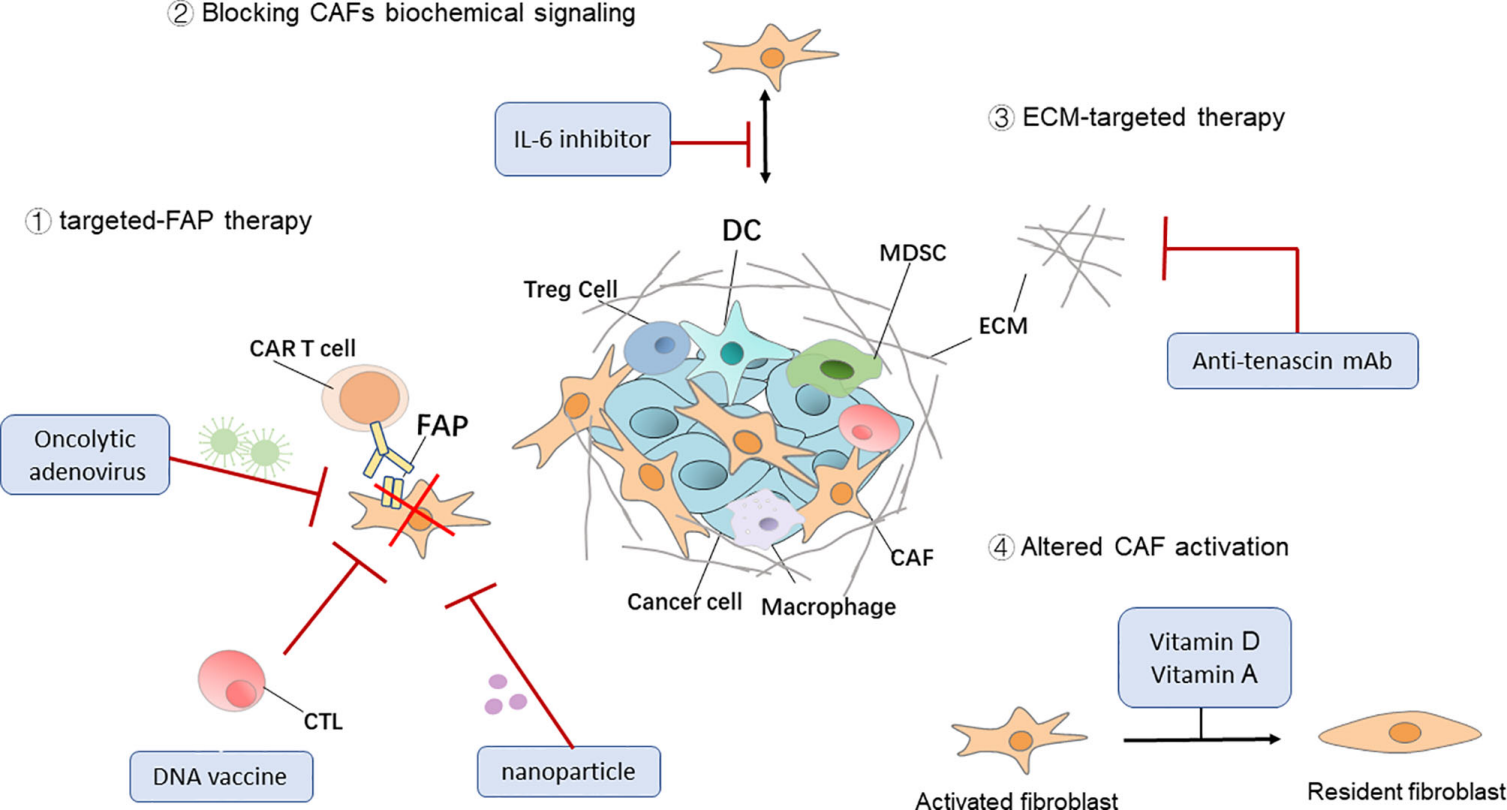
Therapeutic targeting of CAFs. Four general approaches that target cancer-associated fibroblasts (CAFs) for cancer immunotherapy. DC, dendritic cell; Treg cell, regulatory T cell; MDSC, myeloid‐derived suppressor cell; ECM, extracellular matrix; CAR, chimeric antigen receptor.

Although anti-CAF therapies have been primarily focused on FAP, reverting the CAF “state” of the resident fibroblasts, blocking CAF biochemical signaling, and ECM-targeted therapy are other examples of promising CAF-targeted therapies. CAFs are often described as a set of continuously active fibroblast cells that support tumor progression (or in some cases inhibit tumors), so one of the ways to counteract their pathological function is to return CAFs to a dormant or tumor-suppressive state. Deficiencies in fat-soluble vitamins, such as vitamins A and D, are often found in patients with PDAC. In a mouse model of PDAC, CAFs isolated from this tumor model were reduced to an inactivated state after all-trans retinoic acid was used to restore retinol levels and inhibit tumor growth (95). Investigators are also targeting CAF-derived cytokines and chemokines in combination with immunotherapies to inhibit tumor progression. A phase I clinical study (96) demonstrated that a combination of a humanized monoclonal antibody targeting IL-6 with interferon, carboplatin, or doxorubicin is safe and effective for the treatment of ovarian cancer. Tenascin C is a CAF-derived ECM protein that regulates cell adhesion and metastatic activity. Researchers have used tenascin C inhibitor (^131^I-m81C6) in combination with immunotherapies and standard chemotherapies in order to ameliorate ECM stiffness to facilitate drug delivery (97) (**Figure 2** and **Table 2**).

Although fibroblasts are abundant in tumor stroma, they have been neglected in the past few decades. Nowadays, the vital role of CAFs is receiving widespread attention in the field of cancer biology. The therapy of targeted CAFs is an effective way to inhibit cancer progression and metastasis by reducing immunosuppression and remodeling TME (98). However, its mechanism of action in the microenvironment of different tumors is not the same, which needs to be further studied. The further elucidation of the mechanism of action of CAF, the discovery of more specific targets, the development of more stable drugs and the selection of patients are all the difficulties to realize the clinical application of targeted CAF.

## Conclusion and Perspective

By participating in the complex regulation of the TME, CAFs not only promote the proliferation of tumor cells and improve the immunosuppression in the TME, but also contribute to LN metastasis (99), and ultimately cause the occurrence and development of tumors. In recent years, there has been a preliminary understanding of CAF origin, specific markers, interactions with various components in the TME, and mechanisms involved in therapeutic resistance. With the in-depth study of the interaction mechanism between CAFs and immune cells in tumors and the TME, an increasing amount of evidence indicates that CAFs are expected to be targets of anticancer therapy. Researchers are targeting CAF-derived cytokines and chemokines in combination with immunotherapies in an attempt to improve anticancer efficiency (5, 98, 100). Although CAF-targeted therapies have received special attention, their mechanism of action in the microenvironment of different tumors is not the same; therefore, further research is required.

## Author Contributions

Manuscript writing and editing were done by P-YC and W-FW. WW and L-SF supervised this review. All authors contributed to the article and approved the submitted version.

## Funding

This work was supported by the China Postdoctoral Science Foundation (grant number: 2019M662867).

## Conflict of Interest

The authors declare that the research was conducted in the absence of any commercial or financial relationships that could be construed as a potential conflict of interest.

The handling editor declared a past co-authorship with one of the authors WW.

## Abbreviations

CAF, cancer-associated fibroblast; CTL, cytotoxic T lymphocyte; DC, dendritic cell; ECM, extracellular matrix; FAP, fibroblast activation protein; LN, lymph nodes; MDSC, myeloid-derived suppressor cell; PDAC, pancreatic ductal adenocarcinoma; PDGFR, platelet derived growth factor receptor; SDF-1, stromal-derived growth factor-1; α-SMA, α-smooth muscle actin; TME, tumor microenvironment; Treg cell, regulatory T cell; FBiTE, FAP-targeting bispecific T-cell engager.
